# Supplementary material for: Vernalization-triggered expression of the antisense transcript COOLAIR is mediated by CBF genes
Source: eLife. 2023 Feb 1;12:e84594. doi: 10.7554/eLife.84594 (PMC10036118; doi:10.7554/eLife.84594)

Figure 1—source data 1.
Uncropped labeled gel image and the original image file for the EMSA result.
Dotted outline indicates the cropping.

Figure panel (Figure 1B)

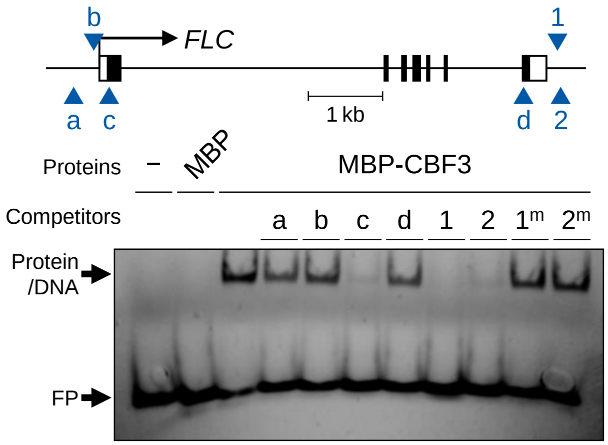

Source data 2

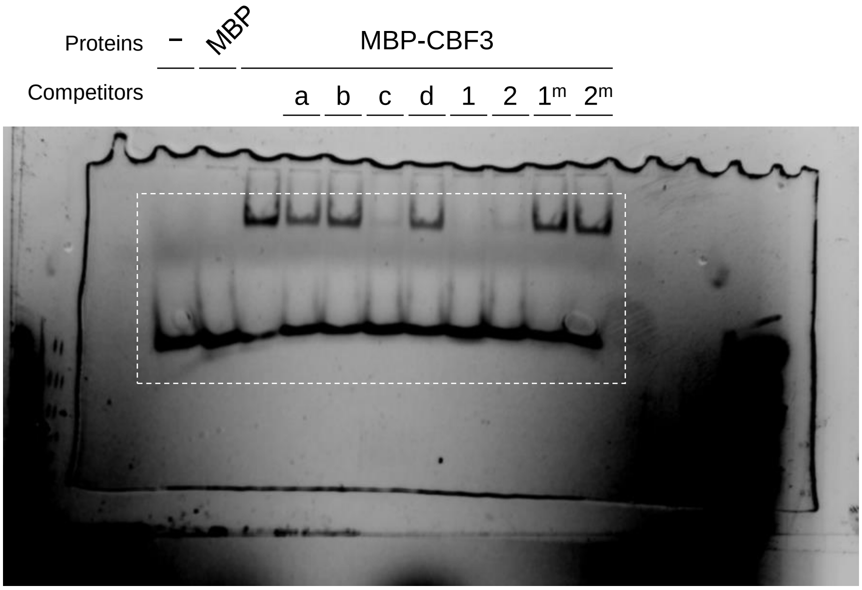

Supplement: Figure 1—source data 1. [file elife-84594-fig1-data1.zip › Figure 1—source data 1.pdf]
