# Supplementary figures and images for: Vernalization-triggered expression of the antisense transcript COOLAIR is mediated by CBF genes
Source: eLife. 2023 Feb 1;12:e84594. doi: 10.7554/eLife.84594 (PMC10036118; doi:10.7554/eLife.84594)

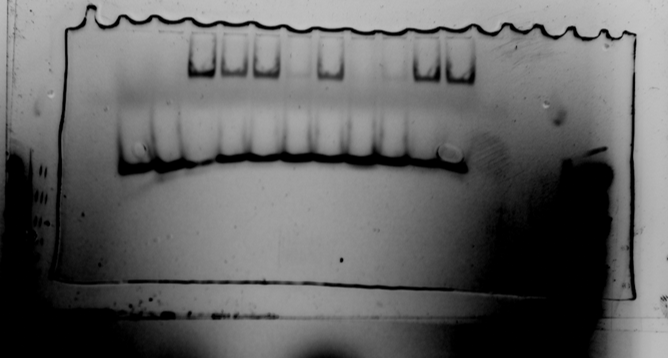

Supplement: Figure 1—source data 1. [file elife-84594-fig1-data1.zip › Figure 1—source data 2.tif]

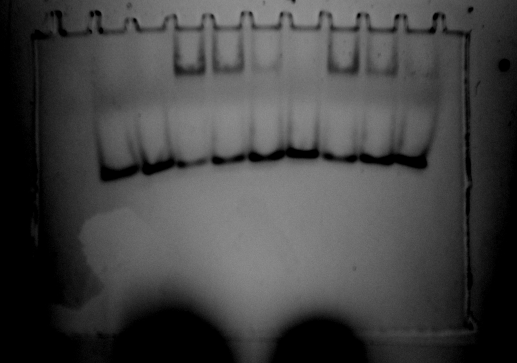

Supplement: Figure 1—figure supplement 2—source data 1. [file elife-84594-fig1-figsupp2-data1.zip › Figure 1—figure supplement 2—source data 2.tif]

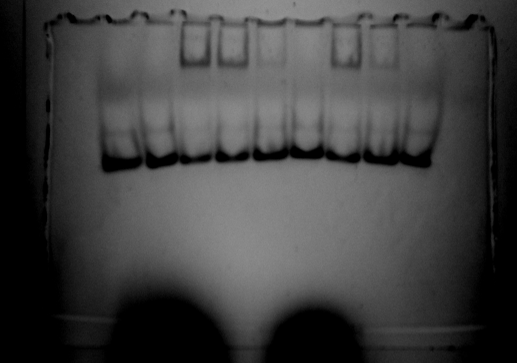

Supplement: Figure 1—figure supplement 2—source data 1. [file elife-84594-fig1-figsupp2-data1.zip › Figure 1—figure supplement 2—source data 3.tif]

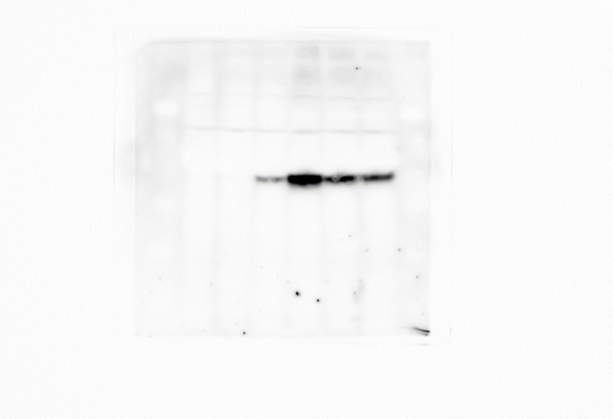

Supplement: Figure 3—source data 1. [file elife-84594-fig3-data1.zip › Figure 3—source data 2.tif]

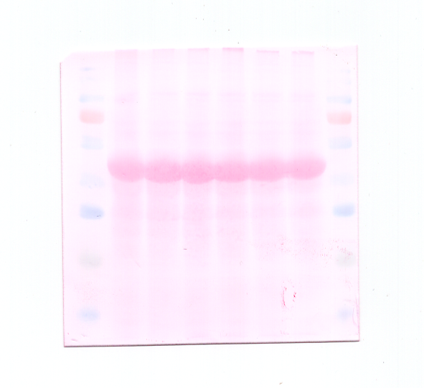

Supplement: Figure 3—source data 1. [file elife-84594-fig3-data1.zip › Figure 3—source data 3.tif]

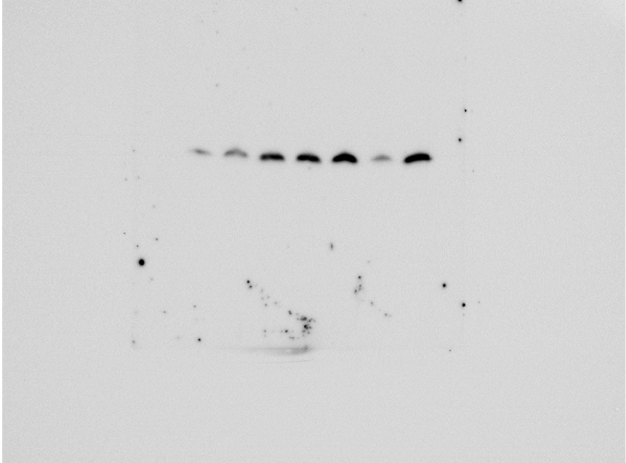

Supplement: Figure 3—source data 1. [file elife-84594-fig3-data1.zip › Figure 3—source data 4.tif]

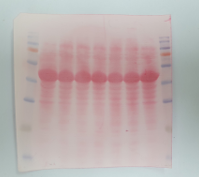

Supplement: Figure 3—source data 1. [file elife-84594-fig3-data1.zip › Figure 3—source data 5.tif]

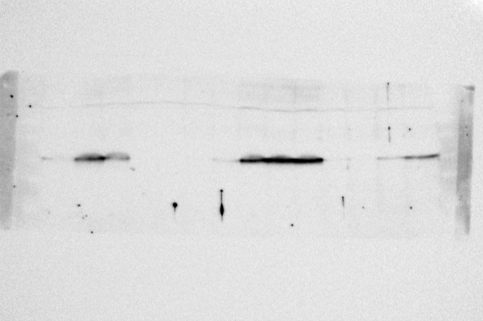

Supplement: Figure 3—figure supplement 1—source data 1. [file elife-84594-fig3-figsupp1-data1.zip › Figure 3—figure supplement 1—source data 2.tif]

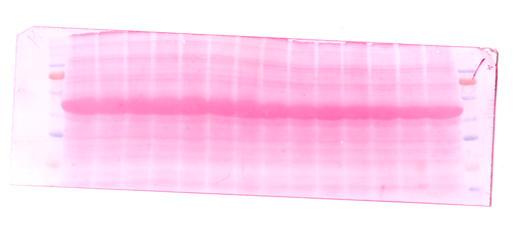

Supplement: Figure 3—figure supplement 1—source data 1. [file elife-84594-fig3-figsupp1-data1.zip › Figure 3—figure supplement 1—source data 3.tif]

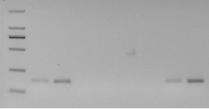

Supplement: Figure 5—source data 1. [file elife-84594-fig5-data1.zip › Figure 5—source data 2.tif]

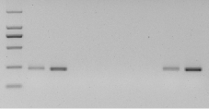

Supplement: Figure 5—source data 1. [file elife-84594-fig5-data1.zip › Figure 5—source data 3.tif]

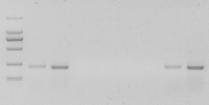

Supplement: Figure 5—source data 1. [file elife-84594-fig5-data1.zip › Figure 5—source data 4.tif]

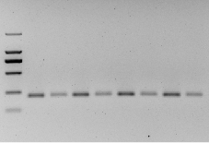

Supplement: Figure 5—source data 1. [file elife-84594-fig5-data1.zip › Figure 5—source data 5.tif]

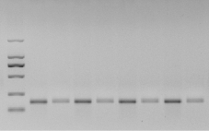

Supplement: Figure 5—source data 1. [file elife-84594-fig5-data1.zip › Figure 5—source data 6.tif]

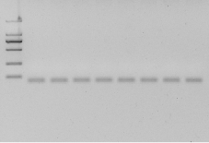

Supplement: Figure 5—source data 1. [file elife-84594-fig5-data1.zip › Figure 5—source data 7.tif]
