# Supplementary material for: Vernalization-triggered expression of the antisense transcript COOLAIR is mediated by CBF genes
Source: eLife. 2023 Feb 1;12:e84594. doi: 10.7554/eLife.84594 (PMC10036118; doi:10.7554/eLife.84594)

Figure 1—figure supplement 2—source data 1.
Uncropped labeled gel images and the original image files for the EMSA results.
Dotted outlines indicate the cropping.

Figure panels
(Figure 1—figure supplement 2B)

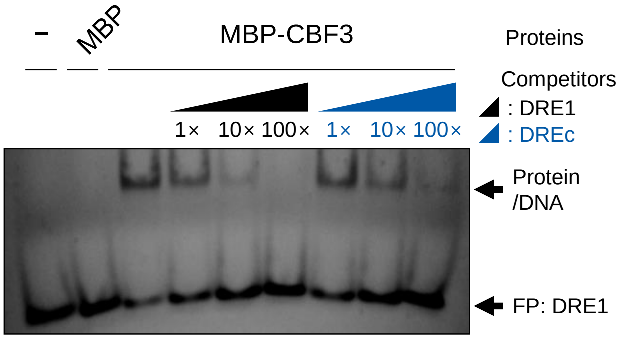

Source data 2

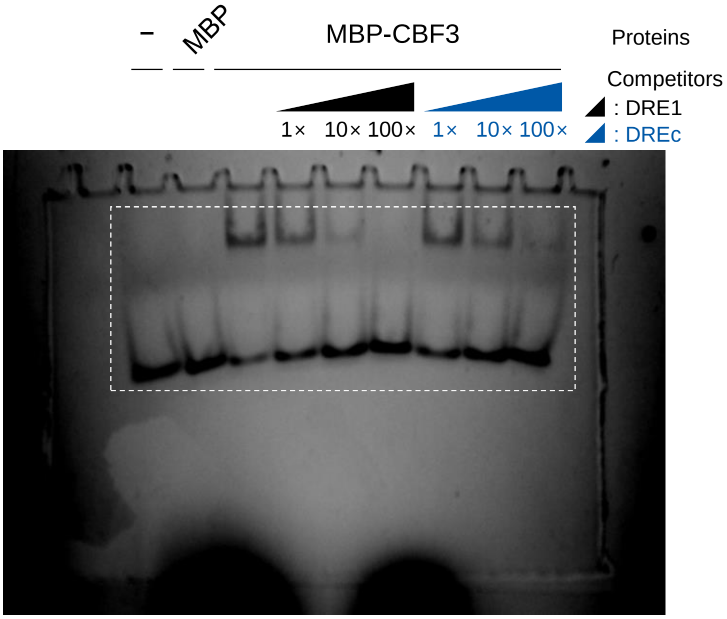

(Figure 1—figure supplement 2C)

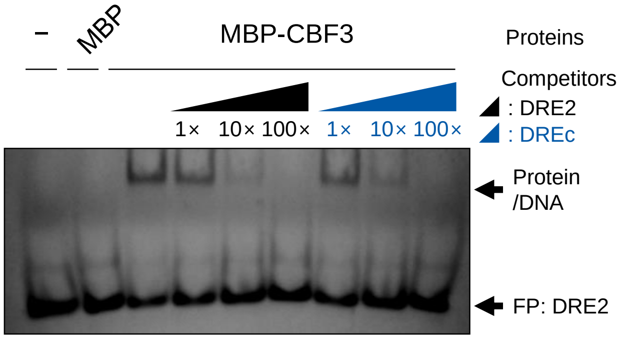

Source data 3

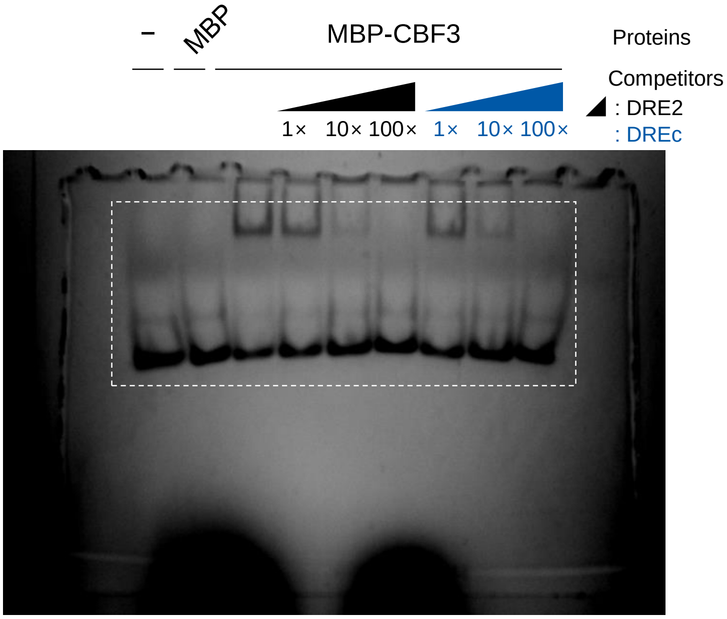

Supplement: Figure 1—figure supplement 2—source data 1. [file elife-84594-fig1-figsupp2-data1.zip › Figure 1—figure supplement 2—source data 1.pdf]
