# Supplementary material for: Vernalization-triggered expression of the antisense transcript COOLAIR is mediated by CBF genes
Source: eLife. 2023 Feb 1;12:e84594. doi: 10.7554/eLife.84594 (PMC10036118; doi:10.7554/eLife.84594)

Figure 3—source data 1.
Uncropped labeled blot images and the original image files for the immunoblots.
Dotted outlines indicate the cropping.

Figure panel (Figure 3D)

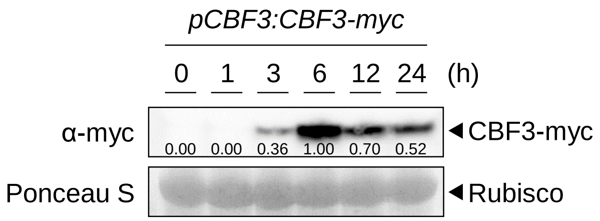

Source data 2
Anti-myc immunoblot (chemiluminescence)

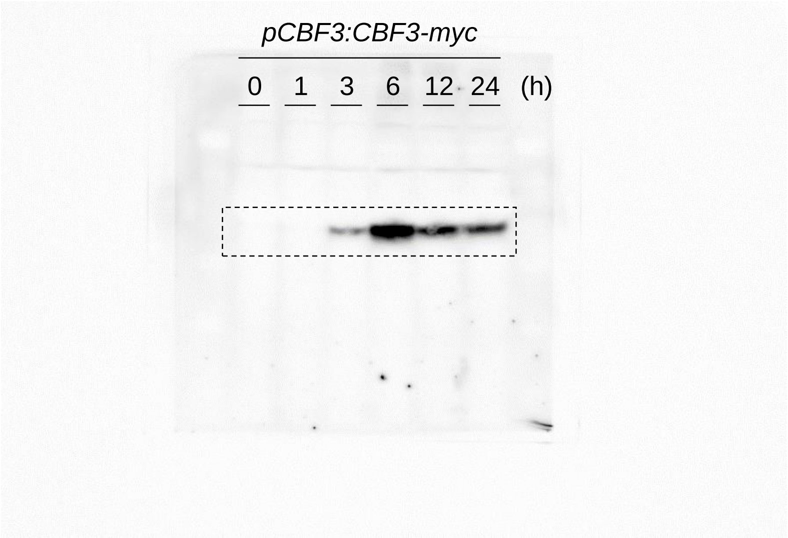

Source data 3
Ponceau S staining

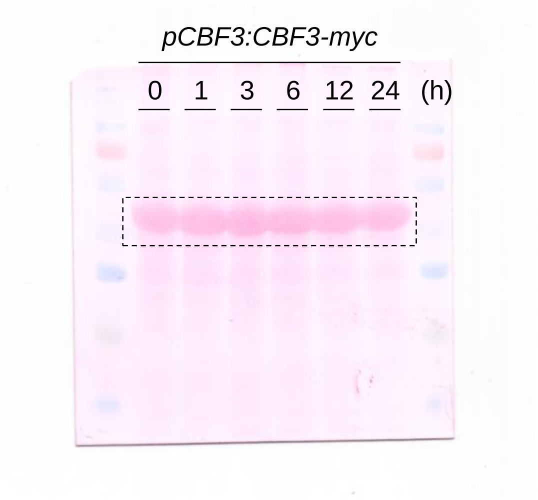

Figure panel (Figure 3E)

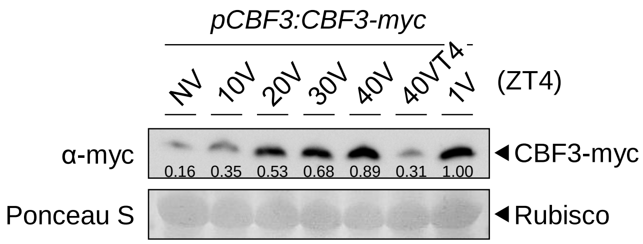

Source data 2
Anti-myc immunoblot (chemiluminescence)

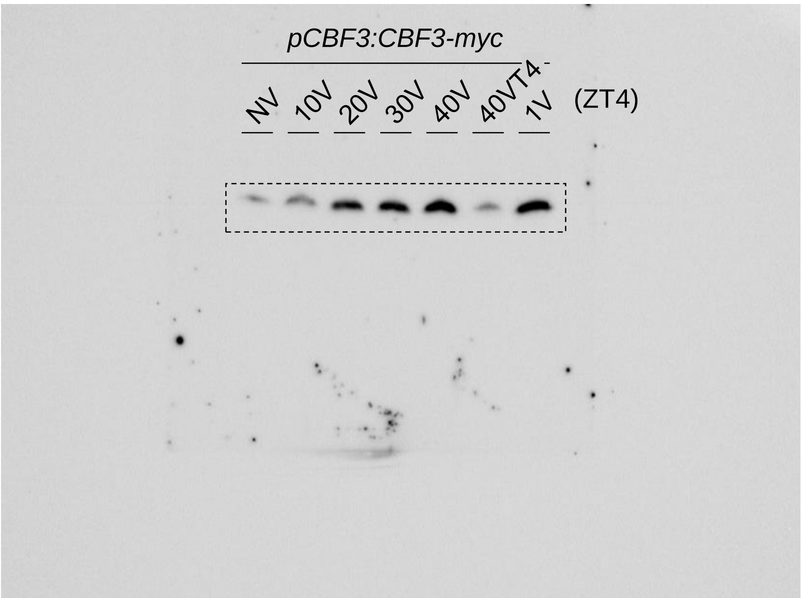

Source data 3
Ponceau S staining

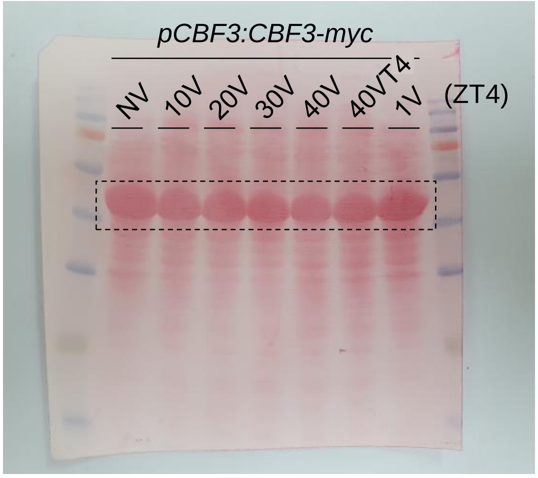

Supplement: Figure 3—source data 1. [file elife-84594-fig3-data1.zip › Figure 3—source data 1.pdf]
