# Supplementary material for: Vernalization-triggered expression of the antisense transcript COOLAIR is mediated by CBF genes
Source: eLife. 2023 Feb 1;12:e84594. doi: 10.7554/eLife.84594 (PMC10036118; doi:10.7554/eLife.84594)

Figure 3—figure supplement 1—source data 1.
Uncropped labeled blot images and the original image files for the immunoblots.
Dotted outlines indicate the cropping.

Figure panel (Figure 3—figure supplement 1)

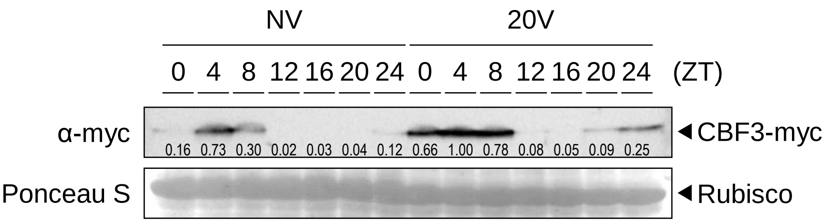

Source data 2
Anti-myc immunoblot (chemiluminescence)

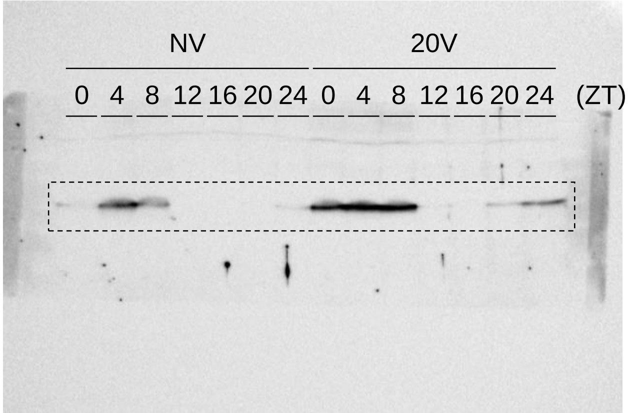

Source data 3
Ponceau S staining

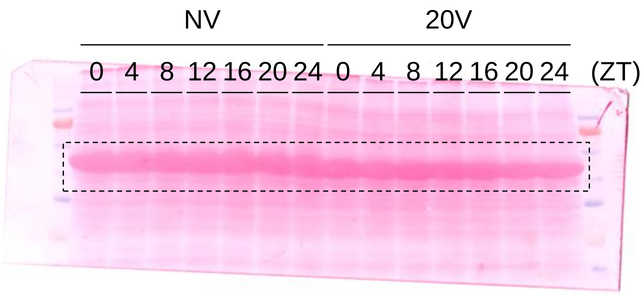

Supplement: Figure 3—figure supplement 1—source data 1. [file elife-84594-fig3-figsupp1-data1.zip › Figure 3—figure supplement 1—source data 1.pdf]
