# Supplementary material for: Vernalization-triggered expression of the antisense transcript COOLAIR is mediated by CBF genes
Source: eLife. 2023 Feb 1;12:e84594. doi: 10.7554/eLife.84594 (PMC10036118; doi:10.7554/eLife.84594)

Figure 5—source data 1.

Uncropped labeled gel images and the original image files for the RT-PCRs.

Dotted outlines indicate the cropping.

Figure panel (Figure 5B)

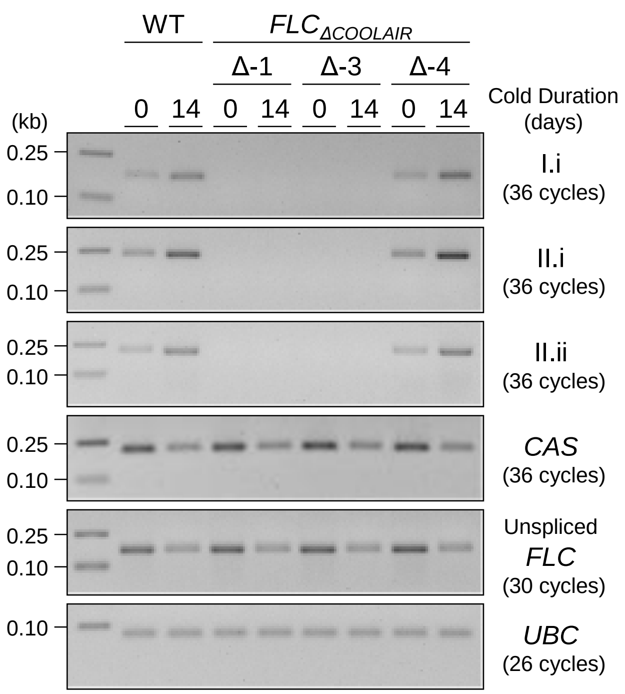

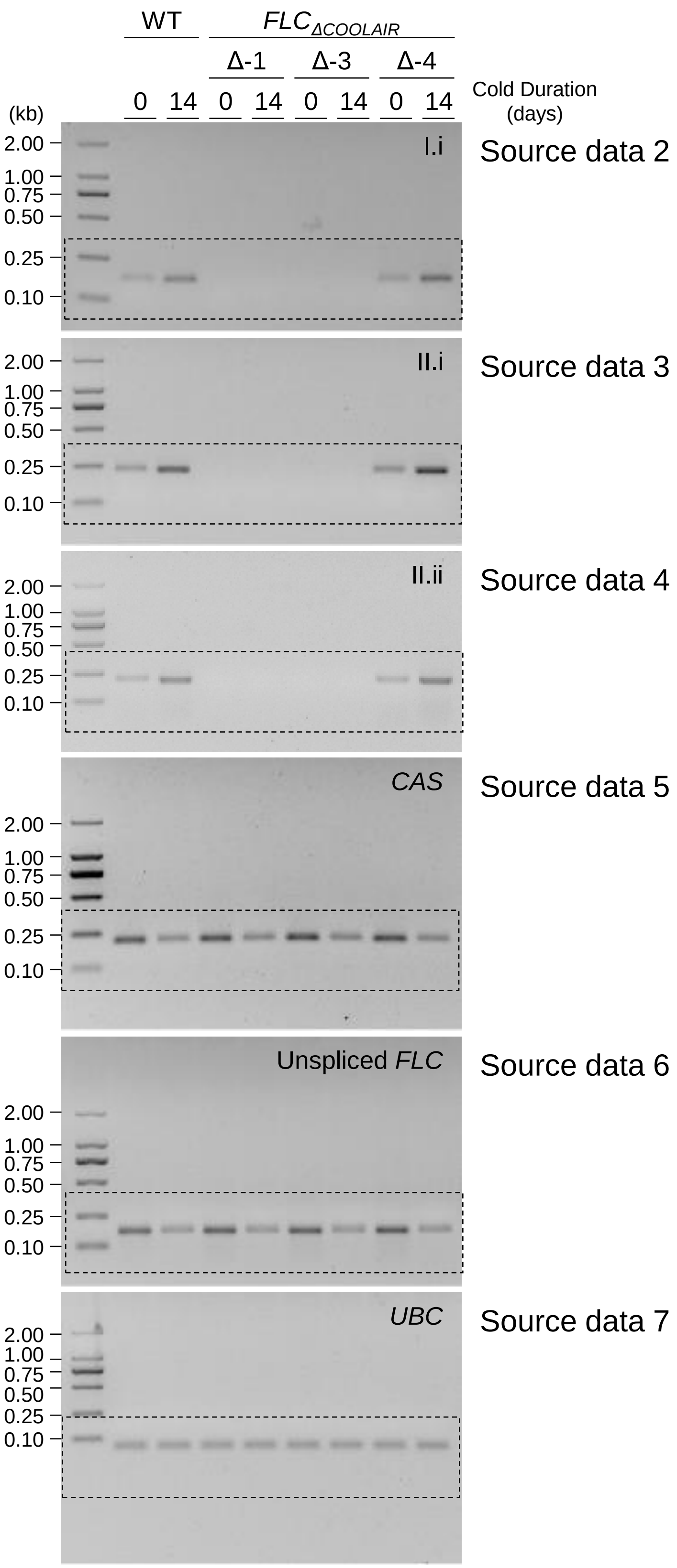

Supplement: Figure 5—source data 1. [file elife-84594-fig5-data1.zip › Figure 5—source data 1.pdf]
